# Achieving Excellence in Graduate Research: A Guide for New Graduate Students

**DOI:** 10.1002/advs.201500203

**Published:** 2015-07-17

**Authors:** Charles B. Parker, Jason J. Amsden, Qing Peng, Brian R. Stoner, Jeffrey T. Glass

**Affiliations:** ^1^ Department of Electrical and Computer EngineeringDuke UniversityDurhamNC27708USA; ^2^ Department of Chemical and Biological EngineeringUniversity of AlabamaTuscaloosaAL35487USA; ^3^ Discovery‐Science‐Technology DivisionRTI InternationalResearch Triangle ParkNC27709USA

**Keywords:** attending conferences, graduate school, mentoring, writing papers

## Abstract

**A mentoring guide for incoming graduate students** has been developed to minimize the time spent reiterating general guidance and “norms” that need to be instilled in new graduate students. This allows principal investigators and senior researchers to provide high value, customized coaching for the individual student which is where the real value of the PhD education is expressed.

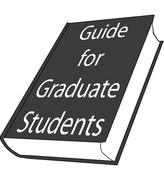

## 1 Introduction

One of the benefits of graduate education for PhD students is the custom mentorship that students receive from senior researchers, including principal investigators, lab managers, and research scientists. It is also one of the most enjoyable activities of being a faculty member. However, too much time is spent reiterating general “norms” that need to be instilled in students (and postdoctoral researchers) rather than providing customized mentorship for the individual which is where the real value of the education is expressed. Thus, the mentoring guide for incoming graduate students below was developed to provide a platform from which new students could begin their acclimation to our research group. As an unexpected benefit, it has also been found to help set student expectations and even provides some assistance in helping students decide if they want to join the group (i.e., is it a good fit for them). This published version has been generalized but without making it too generic. Some of these items are only relevant to students pursuing an experimental degree. Some items are informed by the specific area (i.e., nanomaterials, materials science, electrical engineering, etc.), whereas other topics are broad enough that they will cover most fields. Guidance and suggestions are provided on group philosophy and expectations, writing papers, attending conferences, and professional conduct. This information is provided as a guide for graduate students when they first join a new research group and it has been found to be a useful tool to help students become acclimated to the graduate research environment.

This is an open access article under the terms of the Creative Commons Attribution License, which permits use, distribution and reproduction in any medium, provided the original work is properly cited.

## 2 Group Philosophy and Expectations

### 2.1 Safety First—Always

You are not only obligated to follow safe practices for your own work and in your individual lab area but if you see something you are concerned about, it is your obligation to bring it to the attention of the group. Never, ever compromise safety to get results. An organized work area is as important for safety as following chemical and biological safety protocols.

### 2.2 It Should Be Your Passion

If you are not excited about your research, you need to change something. Your supervisor can help you decide what needs to change but ultimately it is your responsibility. Working long hours and dealing with all the ups and downs that naturally occur in research is very tough if you do not love it. Ultimately, your passion and work effort will determine your graduation date.

### 2.3 It Is a Tough Job

Getting a graduate degree is a demanding job, not a casual student experience. That means it is not just 40 h a week (in today's world, almost no professional job is if you want to be successful). It reportedly takes 10 000 h of focused practice to become an expert and there is always a period of tedium you need to pass through to get there.^1^ Like any entry level job, vacation is limited by the policies of the organization and research group. If you are taking time off, you should have in mind that it needs to fall within this scope. The better your focus and the more hours you put in, the higher the probability you will graduate in a timely manner. And reflecting on the previous point, you will find that the more passion you have about your chosen research, the less it will feel like a “job” and more like fulfilling that passion. In fact, if you are excited and focused about your research then it is likely your supervisor will need to tell you that you should take some time off and find a bit more life balance!

### 2.4 A Sense of Urgency

This is the only way to lead your field. Remember that there is always another group working on the same type of research that you are, so do not wait to publish your work or someone else will publish it first. Many times the difference between getting credit for a significant advance, including the hundreds of citations that can go along with it, and getting very little recognition, can be just a few weeks. This means efficiency and hard work are both required. But remember, NEVER compromise safety or integrity to get results.

### 2.5 Publish or Perish

A key part of the graduate research environment is to disseminate your work through scientific publications. Write papers as you go along, not at the end of your degree. Consider publishing an exciting advance quickly as a letters paper so you are the first to report it and then follow up with a full paper. Remember, it is in your best interest to get as many high‐quality publications as possible from your work, especially if you want to go into academics. However, do not publish trivial, incremental results just to increase the number of your publications. Each paper should tell a complete story of value to other researchers. Publications are also generally a good way to determine if you have completed sufficient research for your dissertation. The question “when can I graduate” is much easier to answer when you think about how many papers you and your advisor expect you to submit before you defend your dissertation.^2^ Every student should also be familiar with ethical guidelines for publishing such as those found in ref.^3^.

### 2.6 Communication Is Critical

During the workday, there should be rapid turnaround on time sensitive e‐mail and immediate (more or less) turn around on time sensitive text messages. This means you should check email at the beginning, middle, and end of every day. It should not interrupt your research. If you utilize multitasking skills, you should be interspersing email between your experiments and it should not slow down the experiments. When you are traveling without access to email, let your team know and put an “Out of Office” auto reply on your email. In general, acknowledging all internal e‐mail that is sent to you within 24 h is good practice unless it is just an informational email sent to the entire group. This helps your teammates know that you received it. If it requires an action, let the sender know when you expect to complete that action.

### 2.7 Idle Time

No one should ever be without something to do. Learn to keep multiple tasks moving in parallel. When one task has you waiting for something, you will have work to do on another. Multitask and generate a running to‐do list. Everyone should keep a calendar of tasks, meetings, events, etc. Everyone should have a research notebook they use to help crystallize/archive ideas and experiments.

### 2.8 Housekeeping

Keep your areas neat, return tools after you use them, and be sure to inform people if you borrow items from their area. The organization and cleanliness of an area is associated with its safe use. Help your group's senior researchers police this in a collegial and positive way.

### 2.9 Team Work

Help each other and share best practices. Everyone needs to pitch in regarding safety and housekeeping for the lab. Do not wait for supervisors to require it, take a leadership role and self‐organize to get it done. The same is true for co‐curricular activities such as working with student chapters of professional societies, student‐led seminars, hosting visitors, etc. And of course, collaborate on research whenever possible. It will make all of the projects better.

In summary:

- Safety First— Always
- It Should be Your Passion
- It Is a Tough Job
- A Sense of Urgency Is Needed
- Publish or Perish
- Communication Is Critical
- Idle Time Should Be Used for Parallel Projects
•Do Not Forget the Housekeeping
- Team Work Is Essential
•Safety First—Always

## 3 Writing Papers

Here is some guidance on writing papers, including optimization of the writing process, how to collaborate while writing a paper with other people, and guidance on how to determine authorship.

### 3.1 Writing the Manuscript

The writing process starts with the quality of your research but even with excellent research results, it can be a difficult process. Following some simple guidelines will enable it to proceed much more smoothly.

- To decide what to publish, you must know the literature. You will want to publish something that is novel and you cannot do that without knowing what has already been published. Furthermore, each paper you read should provide at least one new idea for your research. Finally, you will need to reference the literature in your paper, so that also requires that you know the literature.
- Start with an outline that your key authors review before starting to write your text. Then go to a more detailed outline. Next, go to a more detailed outline with figures. By the time you finish the outline phase, you should have a phrase or topical sentence for each paragraph that will be in the final paper. Remember that part of your job as a writer is to make the reader's job as easy as possible.
•Whitesides has published an excellent paper that describes how to write a scientific manuscript.^4^ Please read it. You do not need to follow it exactly but it is a very good tutorial. Several other guides for writing scientific papers^5^ and creating figures may also be useful.^6^
•The lead author on a paper is ultimately responsible for its content. The lead author is in charge of writing the outline, prose, and soliciting comments and edits from coauthors.
- If more than one person is reviewing drafts of a paper, it is very useful to do the reviews in series so you do not need to process parallel copies of the paper. However, this is not always possible because it is advantageous to publish as soon as possible. In that case, have the person who you think will do the most thorough review look at the paper after you. Be particularly careful about filenames in this case to avoid confusion (see below).
- Considering edits made to the text from your coauthors, review them and if they are OK, just accept them before sending out a new draft for review. Do not leave them in during the next round of review. If you do not agree with them, add a comment about why you disagree and leave the change highlighted in the text. If this goes on during multiple revisions, set a meeting to discuss the issue.
- For comments that are made by a coauthor, address them in the revised paper using tracked changes and simply write in the text box under the coauthor's comment with your name or initials “JTG: Complete.” If the comments require some explanation or if they ask a question, write this under the coauthor's comment, for example: “JTG: This paragraph is the first time we describe this procedure so rather than eliminating it, I eliminated the discussion in the next section.”
- Naming files is critical when collaborating on a paper. Always think about the naming from the perspective of your collaborators, not your own perspective. Using a common method for naming files helps us keep track of who has looked at a document and helps us recover the document if there are problems:
  - This is not helpful: thing‐4 finalfinal v3 (with new changes!) 15b.docx
  - Rather, use a logical filename such as an abbreviation of the topic followed by a version number: Important_paper v01.docx.
  - To edit a paper provided by the primary author, use tracked changes and append the filename with your initials: Important_paper v01‐cbp.doc
  - If you are the primary author on the paper, or it is agreed that you are holding the master copy, after you address the changes and comments from your coauthors, send the next draft with a new version number and delete all of the initials at the end of the filename.
- Although many journals require figures to be in a separate document or at the end of the paper, for the review process you should embed the figures in the text where they are referred to. Put the figures in a table when in Microsoft Word so they do not float around in the text. The caption should go in a separate row or column in the table. The figure numbers in the text should be hyperlinked to the figure captions so they change automatically when new figures are put in. This is very easy in Microsoft Word.
- Remember that authorship can be a tricky issue so do not put authors on an outline without thinking about it first and talking with your primary coauthors/advisor. Please see the guidance on authorship notes in the next section.

### 3.2 Coauthorship

Coauthorship can be a sensitive topic and is not always clear. Guidance on coauthorship can be found in ref.^7^ When in doubt, it is good to be more inclusive but coauthorships should not be given out simply to be nice. Here are some general principles of coauthorship. Note that this is different than inventorship on a patent which is legally defined.^8^

- Inclusion as a coauthor is generally based on substantial contribution to one or more of the following:
  - conception and design of the experimental approach
  - acquisition of data
  - analysis and interpretation of the data
  - drafting/organization of the intellectual content of the article
  - revising the article critically for important intellectual content
- Individuals that provide other inputs without which the manuscript could not have been prepared may also warrant inclusion (i.e., lab support, technical advice, etc.).
•Coauthors are expected to be able to defend some part of the science of the study (i.e., they should not be doing rote procedures provided by someone else such as would be expected of an assistant).
- Individuals that provided services for hire or technical advice may warrant mention in the “Acknowledgements” rather than coauthorship.
- The first named author is ultimately responsible for all aspects of the manuscript. However, all authors should be involved.
•No person should be included as a coauthor without their expressed consent.
- All authors should agree to the specific journal for the submission.^3^
- The corresponding author signs a copyright license on behalf of all the authors.

## 4 Attending a Conference

Presenting your work at conferences and workshops in either an oral or a poster format is important to gain visibility for your research and enable people to get to know you and your work. However, there are other reasons to attend conferences, including, in rough order of priority: i) networking, ii) learning new directions in your field, iii) helping you to think outside your normal boundaries.

### 4.1 Attending Presentations

- Listening to talks is not explicitly listed above but it is part of all of these items. Although it may not be the most important goal, in order to network and learn new directions in your field, especially early in your career, you must attend the talks! Interestingly, it does not really matter how entertaining, well organized, or even scientifically rigorous the talk is, it can still be an entrée into your primary goals for attending the conference.
- Going to talks helps you understand what is happening in the field. Remember that the talks were selected by a program committee so it reflects what they think is important. Even if the talk is not very good, it was chosen to be presented from an abstract and this can be quite meaningful for some conferences. The invited talks also tell you which groups are recognized in the field.
- Just like when reading a paper, most talks you go to should spark an idea with your own work, however small or crazy. You should not expect to actually act on every idea since screening out lower priority ideas so you focus your effort is critical. Nonetheless the exercise of making connections between the talks and your own work is very beneficial and will help exercise your creativity and hone your research skills.

### 4.2 Networking

•Going to talks helps you meet people! It is quite easy to talk to a speaker after a presentation. Let them know what you liked in their presentation and ask a question you are interested in that starts a dialogue, etc. Of course, sometimes the speaker is swamped after a talk and you need to find them at a later, more opportune time during the conference. Even very busy researchers are generally open to a dialogue when you meet them at a conference.
- Networking is about building relationships. You need to cultivate each relationship so it is not just about meeting as many new people as you can. Try to have coffee, lunch, or dinner with someone new each day. Be sure to get their contact information. Walking the line between being proactive and being too pushy is important but most of us need to be more proactive (i.e., get a little closer to the pushy side of the equation). When you get a chance, read a book on networking.^9^
•At most conferences, the activities that are going on outside the talks are extensive and taking advantage of them is important. From receptions to poster sessions to social activities to conference administration; all of them afford the opportunity to get to know the field and people in the field. Impromptu meetings with alumni from your alma maters and unexpected corporate receptions can occur by fortuitously meeting former peers or colleagues. These activities are also a great way to network for a possible job.
- When you meet people, follow up with some correspondence and send them your recent publications if it is appropriate (for example, if you discussed the work or just think they might have an interest).
- Going to conferences is easier if you prepare and it gets easier with experience. Even going to the conference of a new society or topic can be challenging the first time because they all have their own way of doing things. By reading as much as possible about the venue and the sessions before hand, it can really ease the burden while you are there. Look for talks and posters that fit your research and mark them. Most conferences have online apps to make this easier.
- It can get tiring listening to talks all day for several days. Feel free to pick and choose the best talks for your interests from the abstracts. But do not forget to look for interesting talks in peripheral areas that may not look like a good fit on the surface or may not be in the typical symposium you attend within the conference but may still report on a technique or a material system that is of interest. You should solicit requests to attend and report on talks from people in your lab who are not going to the conference. This will give you an expanded purpose and opportunity for some personal growth at each conference.
- Consider being ready to give an oral presentation when you attend a conference as a poster presenter or just as an attendee in case there is a cancellation. It is not uncommon for a presenter to be a no‐show and the session chair to solicit people just before the session to fill the empty time slot (usually on a “first‐come first‐served” basis).

### 4.3 Presenting at a Conference

- Watch other presenters in the same room you will be in to get an understanding of how they are using the space and AV equipment.
- On the break, check out the podium area, the microphone, the laser pointer, etc., that you will be using.
- Be sure to load your slides as directed by the conference organizers (read all instructions, many times the chair of the session will not know all the details). Sometimes you just plug your computer in and other times you must have your slides on a USB memory stick.
- Have a back‐up of your talk in a different format and in two places: on your laptop and on a memory stick in both PDF (which is good as a fallback format) and PowerPoint. If there is someone else from your group at the presentation, ask them to have a back‐up of your talk on their laptop.
- Check your slides in the presentation room if one is available before the session or on a lunch break in the actual room where you will be presenting.
- Do not expect that you will have internet access where you are presenting.
- Check in with the Chairperson of your session right before the session starts to let them know you are present and confirm you are in the right place (and to network!).
- There is nothing that takes the place of practicing your talk out loud several times before you present. Even experienced presenters still do this when presenting new material.
- Always start with something easy like thanking the session organizers.
- In a poster session, be proactive about asking people if they would like you to explain your poster. Allow them to review on their own if they prefer.

## 5 Professionalism

Professionalism in the workplace is a big topic. It is situation dependent and requires significant self‐awareness. This document can only scratch the surface but here are some of the important issues you should consider. As with many of the topics in this document, self‐study is encouraged:

- Do not over commit
•Maintain a proactive and positive attitude
- Identify and help resolve problems
- Dress for the occasion; or a little nicer than the occasion
- Network, meet people and build relationships
- When in doubt, quality over quantity
- Respect for others; respect for diversity
•Cultivate integrity (honesty; sincerity; trustworthiness)
- Be open, direct, and forthright
- Be punctual; if late to a commitment, let someone know
- Develop self‐awareness—learn your strengths/weaknesses and how you impact others

## 6 Conclusion

In conclusion, there are many expectations and organizational norms of behavior for new graduate students. These should be communicated as clearly and as early as possible when a graduate student joins a new research group. In this guide, an attempt has been made to capture many of those that are likely to be similar across different groups and institutions. If graduate students can learn these norms quickly and without requiring significant management time, their interactions with senior members of the lab and advisors can be focused on more subtle and detailed scientific exchanges as well as customized professional development discussions.
